# Long-term rates of change in musculoskeletal aging and body composition: findings from the Health, Aging and Body Composition Study

**DOI:** 10.1007/s00223-020-00679-2

**Published:** 2020-03-03

**Authors:** Leo D. Westbury, Holly E. Syddall, Nicholas R. Fuggle, Elaine M. Dennison, Jane A. Cauley, Eric J. Shiroma, Roger A. Fielding, Anne B. Newman, Cyrus Cooper

**Affiliations:** 1 MRC Lifecourse Epidemiology Unit, University of Southampton, Southampton, UK; 2 Victoria University of Wellington, Wellington, New Zealand; 3 Department of Epidemiology, Graduate School of Public Health, University of Pittsburgh, Pittsburgh, USA; 4 Laboratory of Epidemiology and Population Sciences, Intramural Research Program, National Institute on Aging, Baltimore, USA; 5 Nutrition, Exercise Physiology, and Sarcopenia Laboratory, Jean Mayer USDA Human Nutrition Research Center on Aging, Tufts University, Boston, USA; 6 NIHR Southampton Biomedical Research Centre, University of Southampton and University Hospital Southampton NHS Foundation Trust, Southampton, UK; 7 NIHR Oxford Biomedical Research Centre, University of Oxford, Oxford, UK

**Keywords:** Epidemiology, Sarcopenia, Osteoporosis, Frailty, Muscle

## Abstract

Musculoskeletal disorders are common among older people. Preventive strategies require understanding of age-related changes in strength, function and body composition, including how they interrelate. We have described, and examined associations between, 9-year changes in these parameters among 2917 Health, Aging and Body Composition Study participants (aged 70–79 years). Appendicular lean mass (ALM), whole body fat mass and total hip BMD were ascertained using DXA; muscle strength by grip dynamometry; and muscle function by gait speed. For each characteristic annualised percentage changes were calculated; measures of conditional change (independent of baseline) were derived and their interrelationships were examined using Pearson correlations; proportion of variance at 9-year follow-up explained by baseline level was estimated; and mean trajectories in relation to age were estimated using linear mixed models. Analyses were stratified by sex. Median [lower quartile, upper quartile] annual percentage declines were grip strength (1.5 [0.0, 2.9]), gait speed (2.0 [0.6, 3.7]), ALM (0.7 [0.1, 1.4]), fat mass (0.4 [− 1.1, 1.9]) and hip BMD (0.5 [0.0, 1.1]). Declines were linear for ALM and accelerated over time for other characteristics. Most conditional change measures were positively correlated, most strongly between ALM, fat mass and hip BMD (*r* > 0.28). Proportion of variation at follow-up explained by baseline was lower for grip strength and gait speed (39–52%) than other characteristics (69–86%). Strength and function declined more rapidly, and were less correlated between baseline and follow-up, than measures of body composition. Therefore, broader intervention strategies to prevent loss of strength and function in later life are required as those targeting body composition alone may be insufficient.

**Electronic supplementary material:**

The online version of this article (10.1007/s00223-020-00679-2) contains supplementary material, which is available to authorized users.

## Introduction

Musculoskeletal disorders are common among older people and are a leading cause of morbidity worldwide [1]. Sarcopenia, the loss of muscle mass and strength with age, is associated with increased risk of disability and mortality and significant healthcare costs [2–4]. Annual direct medical costs attributable to sarcopenia were estimated at $18.5 billion in the United States in 2000 [5] and annual excess health and social care costs associated with muscle weakness were estimated at £2.5 billion in a recent UK study [6]. Although there is no consensus definition of sarcopenia, most criteria use measures of grip strength, gait speed and lean mass. Sarcopenia is now regarded as a disease according to the International Classification of Diseases [7].

Another common musculoskeletal disorder is osteoporosis, characterised by low bone density and micro-architectural deterioration of bone tissue, which increases bone fragility [8]. Osteoporotic fractures are associated with increased risk of morbidity and mortality [9], resulting in huge individual and societal costs; the economic burden associated with osteoporotic fractures was estimated at €37.4 billion in the European Union in 2010 [10].

In addition to declines in lean mass and bone density, age-related changes in body composition include increases in fat mass, particularly in the abdominal area [11]. In general, increased fat mass in older age is related to increased risk of physical disability [12] and all-cause mortality [13], although some protective effects of being moderately overweight (25 ≤ body mass index [BMI] < 30) have been reported in relation to mortality in meta-analyses [14, 15]. However, large studies accounting for smoking and diseases causing weight loss have identified obesity (BMI ≥ 30) and central-adiposity, indicated by greater waist-to-hip ratio, as risk factors for mortality, coronary heart disease and type 2 diabetes [16, 17]. Age-related reductions in lean mass in combination with increases in fat mass can result in the development of sarcopenic obesity, resulting in more adverse health effects than sarcopenia or obesity in isolation [18].

Preventive strategies for musculoskeletal disorders require a better understanding of age-related changes in muscle strength, physical function and body composition (including bone) as well as how these changes interrelate; from a public health perspective, this knowledge could inform the development of interventions to delay adverse changes in musculoskeletal aging. Previous research has established the following: aging is associated with declines in muscle mass, strength, physical function and bone density [19]; declines are greater for muscle strength and physical function compared to muscle mass [20, 21]; and changes in some muscle and bone parameters are correlated [22]. However, to our knowledge, no studies have explored changes in both key sarcopenia components (muscle mass, strength and function) and aspects of body composition (muscle mass, fat mass and bone density) among a single cohort of older people in whom parameters have been measured at multiple time-points. To address this, we have described, and examined associations between, changes in musculoskeletal and body composition parameters among participants in the Health, Aging and Body Composition (Health ABC) Study, USA.

## Methods

### The Health, Aging and Body Composition Study

The Health ABC Study comprises 3075 US men and women (aged 70–79 years at baseline) who were recruited in 1997–1998. A random sample of white and of all of the black Medicare beneficiaries from around Memphis (Tennessee) and Pittsburgh (Pennsylvania) was obtained. Sampled participants received a mailing followed by a telephone eligibility screen. Individuals reporting no difficulty in walking one quarter of a mile or climbing 10 stairs were eligible. Individuals with the following characteristics were excluded: inability to communicate with the interviewer; clear cognitive impairment; having a life-threatening illness or difficulties with activities of daily living (ADL); requiring a walking aid; having an intention of moving outside the area within three years; or currently enrolled in a lifestyle intervention trial. Written, informed consent was provided by all participants and the study was approved by the institutional review boards at the University of Tennessee and the University of Pittsburgh.

### Ascertainment of Participant Characteristics

The study methodology has been described in detail previously [23]. In brief, at baseline (Year 1), sex, race, educational attainment, and health behaviours such as smoking status and alcohol consumption were self-reported using questionnaires. Height and weight were measured using a Harpenden Stadiometer (Holtain Ltd, Crosswell, UK) and a standard balance beam scale, respectively. Physical activity was calculated using an instrument derived from the Leisure Time Physical Activity Questionnaire [24]. Approximate metabolic equivalent unit values were assigned to reported activities and intensity levels to derive caloric expenditure in kcal/kg/h [25]. Total kilocalories expended per week in stair climbing, walking and exercise activity, calculated by multiplying caloric expenditure by the participant's weight (kg), was used as a measure of physical activity. Participants with < 1000 kcal/week were regarded as having low physical activity [26]. Participants were asked whether a doctor had ever told them that they had various medical conditions. For this analysis, number of comorbidities was calculated out of the following: stroke, diabetes, Parkinson’s disease, chronic obstructive pulmonary disease, heart attack or myocardial infarction, congestive heart failure and hypertension.

At Year 2, dietary intake over the previous year was assessed using a nurse-administered food frequency questionnaire (FFQ) comprising 108 items. To assess the extent to which Health ABC participants’ diets conformed to recommendations of the Dietary Guidelines for Americans of 1995 and the Food Guide Pyramid of 1992, a healthy eating index (HEI), ranging from 0 to 100, was calculated for each participant; higher scores reflected healthier diets [27]. Poor diet quality was defined as scores < 51 [28]. More information on the components of this HEI has been published previously [28].

Grip strength was measured two times for each hand at Years 1, 2, 4, 6, 8 and 10 using a Jamar dynamometer according to a standardised protocol throughout all stages of follow-up; maximum grip strength at each time-point was used for analyses. Grip strength values were set to missing for participants with severe hand pain or recent surgery. The calibration of the dynamometers was checked regularly. Customary gait speed was ascertained at Years 2, 3, 4, 5, 6, 8 and 10 by asking participants to walk at their normal speed down a corridor over a total distance of 20 m. Whole-body dual-energy X-ray absorptiometry scans (Hologic QDR 4500A; Hologic, Bedford, MA, USA) were performed at Years 1, 2, 3, 4, 5, 6, 8 and 10 and used to ascertain whole body fat and appendicular lean mass (ALM) in kg. Total hip BMD, a repeatable measurement that is predictive of future fracture, was measured using the same device at Years 1, 3, 5, 8 and 10. The reproducibility and validity of this scanner has been previously reported [29, 30]. Regular DXA phantom scans were performed for quality control and calibration purposes.

### Statistical Methods

Baseline participant characteristics were described using means and standard deviations (SD), medians and interquartile ranges, or frequency and percentage distributions as appropriate. Normality of the following five characteristics was confirmed through visual inspection of histograms: grip strength, gait speed, ALM, whole-body fat mass and hip BMD. The statistical methods that were applied to each of these five characteristics are stated below. Firstly, percentage change since baseline was calculated at each time-point and person-specific linear regression models were used to model percentage change since baseline on age at each time-point; a participant’s estimated annual percentage change is given by the regression coefficient for age. Pairwise comparisons of the mean and variance for each characteristic were performed using t-tests and variance ratio tests, respectively. Second, conditional change (independent of baseline) was characterised by obtaining the residuals from sex-specific linear regression models for characteristics at follow-up (Year 10) on baseline characteristics with adjustment for individual follow-up duration. The proportion of variance at Year 10 that was explained by baseline level and conditional change was estimated. Relationships between conditional change measures, including conditional change in weight from baseline to follow-up, were examined using Pearson correlations. Finally, the mean trajectory in relation to age was estimated using linear mixed effects models with random intercepts and slopes; quadratic and cubic age terms were included as fixed effects if significant (*p* < 0.05).

All analyses were stratified by sex and based on the sample of 2917 Health ABC participants with data on at least one of the characteristics (grip strength, gait speed, ALM, whole-body fat mass and hip BMD) at two or more time-points. Sensitivity analyses included stratification by race as well as sex and, for each characteristic, a comparison of mean trajectories from participants with observations at all time-points as opposed to a minimum of two time-points. Analyses were conducted using Stata, release 15 (StataCorp, College Station, TX, USA).

## Results

### Baseline Participant Characteristics

Baseline participant characteristics among the analysis sample of 2917 Health ABC participants according to sex and race are presented in Table 1. Mean and standard deviation (SD) for age was 74.1 (2.9) years. A higher proportion of men were current drinkers and had post-secondary education compared to women (*p* < 0.001); prevalence of current smoking did not differ by sex (*p* = 0.29). Among both sexes, a higher proportion of black participants were current smokers but a lower proportion were current drinkers compared to white participants (*p* ≤ 0.001); having a post-secondary education was less common among black participants (*p* < 0.001). Regarding the musculoskeletal measures of interest, women had higher fat mass but all other measures were greater among men (*p* < 0.001 for all associations). Although both black men and women had slower gait speed compared to their white counterparts and black men had lower fat mass, the remaining measures were greater among black participants.

**Table 1:** Baseline participant characteristics according to sex and race

Characteristic [Mean (SD) or *N* (%)]	Men			Women		
	White (*n* = 907)	Black (*n* = 511)	All (*n* = 1418)	White (*n* = 823)	Black (*n* = 676)	All (*n* = 1499)
Age (years)	74.4 (2.9)	74.0 (2.7)	74.2 (2.8)*^†^	74.1 (2.8)	73.8 (2.9)	74.0 (2.9)*^†^
Height (m)	1.74 (0.06)	1.73 (0.07)	1.73 (0.07)*	1.59 (0.06)	1.60 (0.06)	1.60 (0.06)*
Weight (kg)	81.4 (12.4)	81.3 (14.3)	81.4 (13.1)*	66.1 (12.1)	75.7 (15.8)	70.4 (14.6)*^†^
BMI (kg/m^2^)	27.0 (3.7)	27.1 (4.3)	27.0 (3.9)*	26.0 (4.5)	29.7 (5.9)	27.6 (5.5)*^†^
Current smoker	45 (5.0%)	106 (20.7%)	151 (10.7%)^†^	59 (7.2%)	83 (12.3%)	142 (9.5%)^†^
Current drinker	582 (64.5%)	233 (46.0%)	815 (57.8%)*^†^	432 (52.6%)	204 (30.2%)	636 (42.5%)*^†^
Low physical activity	468 (51.6%)	373 (73.0%)	841 (59.3%)*^†^	608 (73.9%)	560 (82.8%)	1168 (77.9%)*^†^
Poor diet quality^α^	46 (5.3%)	63 (14.0%)	109 (8.2%)^†^	39 (5.0%)	49 (8.3%)	88 (6.4%)^†^
Number of comorbidities^+^	1 (0, 1)	1 (0, 2)	1 (0, 1)^†^	0 (0, 1)	1 (0, 1)	1 (0, 1)^†^
Post-secondary education	546 (60.3%)	134 (26.3%)	680 (48.0%)*^†^	395 (48.1%)	181 (26.9%)	576 (38.6%)*^†^
Grip strength (kg)	39.7 (7.7)	42.8 (8.7)	40.8 (8.2)*^†^	23.6 (5.1)	26.6 (6.2)	25.0 (5.8)*^†^
Gait speed (m/s)	1.23 (0.19)	1.10 (0.20)	1.19 (0.21)*^†^	1.16 (0.19)	1.01 (0.20)	1.09 (0.21)*^†^
ALM (kg)	23.3 (3.2)	25.0 (3.9)	23.9 (3.6)*^†^	15.3 (2.4)	18.2 (3.2)	16.6 (3.1)*^†^
Fat mass (kg)	24.7 (6.9)	23.2 (7.4)	24.2 (7.1)*^†^	27.0 (7.9)	31.6 (10.2)	29.1 (9.3)*^†^
Hip BMD (g/cm^2^)	0.94 (0.14)	1.02 (0.15)	0.97 (0.15)*^†^	0.77 (0.13)	0.86 (0.15)	0.81 (0.15)*^†^

*SD* standard deviation, *ALM* appendicular lean mass, *BMD* bone mineral density

*Statistically significant sex differences (*p* < 0.05)

^†^Statistically significant racial differences within sex (*p* < 0.05)

^α^Ascertained at Year 2

^+^Median (lower quartile, upper quartile) number of the following conditions (ever told by doctor): stroke, diabetes, Parkinson’s disease, chronic obstructive pulmonary disease, heart attack or myocardial infarction, congestive heart failure and hypertension

Compared to the 158 participants who were not included in the analytical sample, both men and women in the analytical sample were more likely to be white and have post-secondary education (*p* < 0.004); mean baseline grip strength was higher among men (*p* = 0.003) but there were no significant differences in the remaining musculoskeletal and body composition parameters at baseline among men or women.

### Percentage Change in Characteristics

**Fig. 1:**
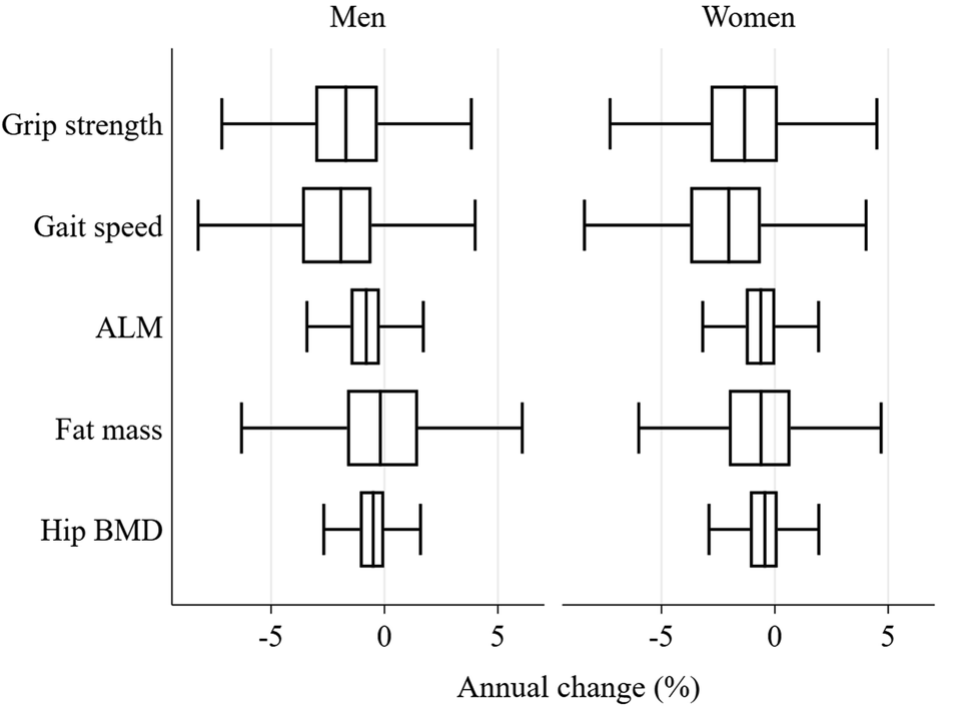
Estimated annual percentage change in characteristics among men and women. Median annual changes (%): grip strength − 1.5, gait speed − 2.0; ALM − 0.7; fat mass − 0.4; hip BMD − 0.5. *ALM* appendicular lean mass, *BMD* bone mineral density. The three vertical lines in the box represent the lower quartile (Q1), median and upper quartile (Q3). The lower whisker is the smallest value that is greater than Q1 − 1.5 × IQR and the upper quartile is the largest value which is less than Q3 + 1.5 × IQR, where IQR = Q3–Q1. Estimates of percentage change for each participant were derived using person-specific linear regression models for percentage change since baseline calculated at each time-point as the outcome with age at each time-point as the only predictor. Annual percentage change is given by the regression coefficient for age. Analysis was restricted to 1418 men and 1499 women with data on at least one change measure

Boxplots of estimated annual percentage change in each characteristic are shown in Fig. 1. Among both men and women, mean percentage declines in grip strength and gait speed were greater than for all other characteristics (*p* < 0.02 for all comparisons). Furthermore, variation in annual percentage decline in grip strength, gait speed and fat mass was greater than for ALM and hip BMD (*p* < 0.001 for all comparisons according to variance ratio tests).

### Mean Trajectories of Characteristics

**Fig. 2:**
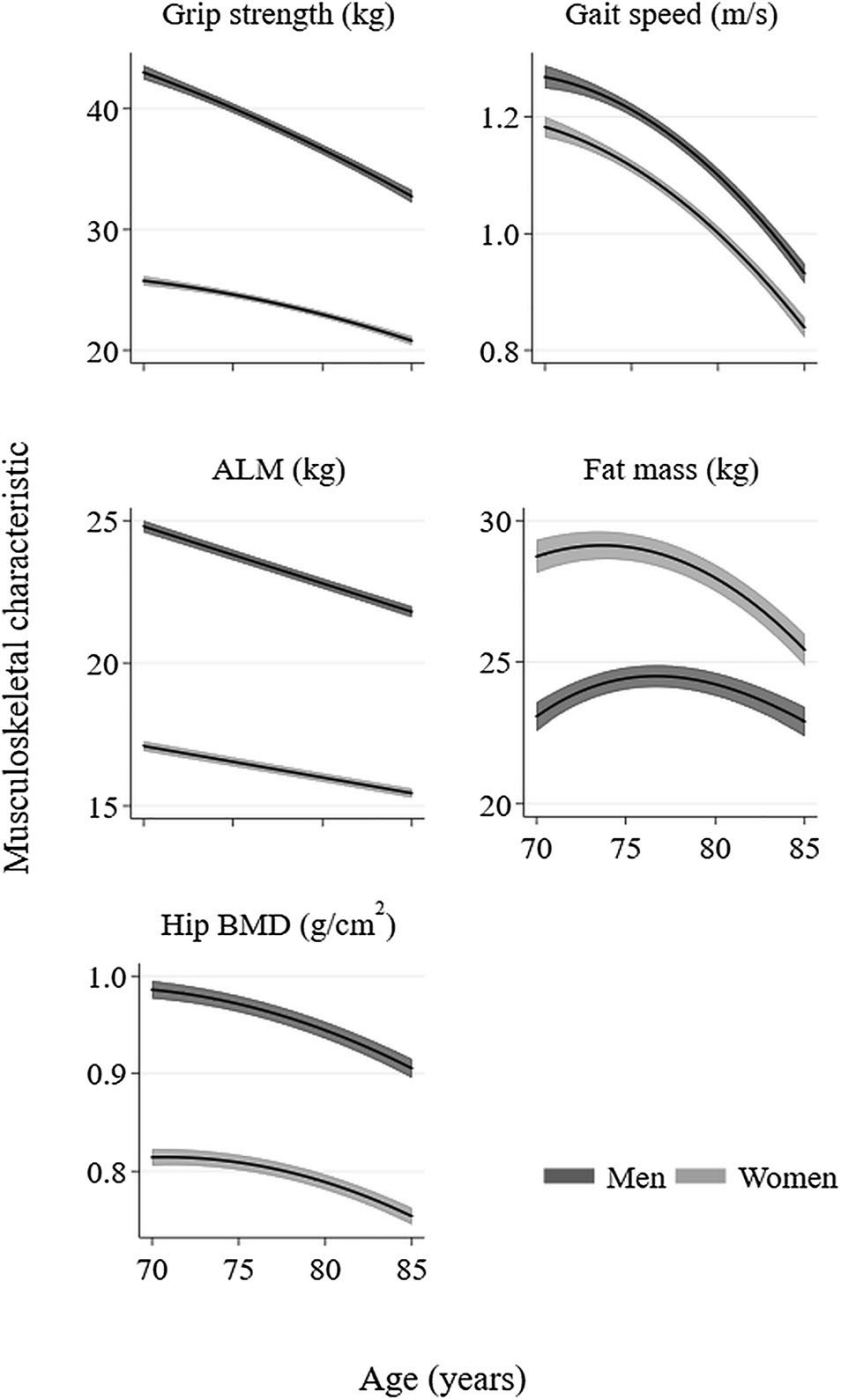
Mean (95% CI) trajectories of characteristics among men and women. *ALM* appendicular lean mass, *BMD* bone mineral density. Mean trajectories were derived using linear mixed effects models with random intercepts and slopes. Quadratic and cubic age terms were included as fixed effects if significant (*p* < 0.05). For each characteristic, trajectories from participants with at least two observations were included

Mean trajectories of characteristics for men and women are presented in Fig. 2. Decline in grip strength, gait speed and hip BMD accelerated somewhat with age; decline in ALM was linear. Fat mass increased, plateaued and then decreased among men, whereas the initial period of increase was negligible among women.

### Proportion of Variation in Characteristics at Follow-Up Explained by Baseline Level and Conditional Change

**Fig. 3:**
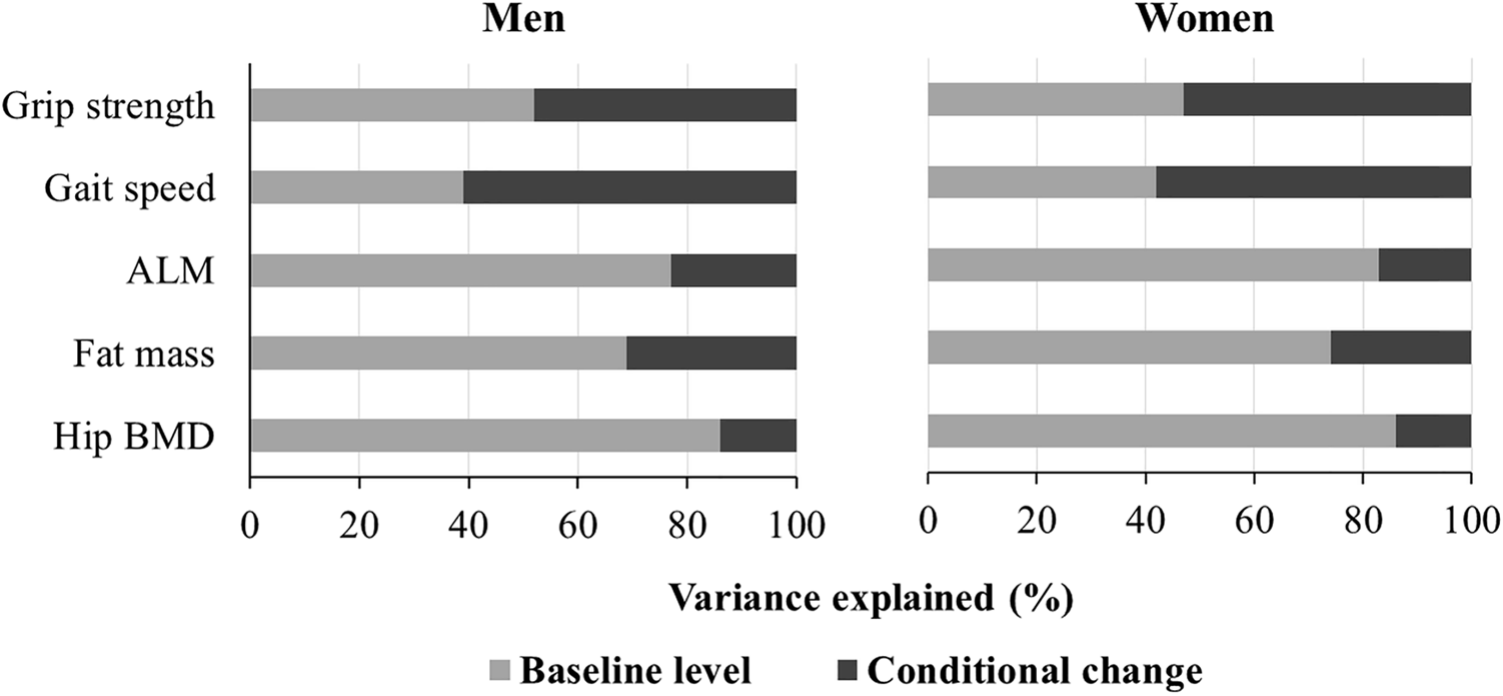
Proportion of variance at follow-up (Year 10) explained by baseline level and conditional change since baseline. *ALM* appendicular lean mass, *BMD* bone mineral density. Measures of conditional change were derived using a residual change method and were independent of baseline level. Analyses restricted to 735 men and 864 women with data on at least one conditional change measure

The proportion of variation in each characteristic at follow-up (Year 10) which was explained by baseline level and conditional change is shown in Fig. 3. Among both sexes, a substantial proportion of the variation (48–61%) in grip strength and gait speed at Year 10 was explained by conditional change since baseline, whereas for other characteristics, this figure was only 14–31%. Equivalently, the correlations between baseline and follow-up measurements were lower for grip strength and gait speed in comparison with the other characteristics.

### Correlations Between Changes in Characteristics

Pearson correlations between conditional change measures for each characteristic are presented in Table 2; most were significantly positively correlated (*p* < 0.05), suggesting that declines in muscle strength, physical function and body composition parameters tend to co-occur. Changes in ALM, fat mass and hip BMD were correlated with one another among both sexes (*r* > 0.28 for all correlations); correlations between changes in fat mass and hip BMD were stronger among women (*r* = 0.47) than men (*r* = 0.29). Changes in grip strength and ALM were moderately correlated among men (*r* = 0.29, *p* < 0.001) and women (*r* = 0.20, *p* < 0.001). Among men and women, changes in weight and fat mass were very highly correlated (*r* > 0.89, *p* < 0.001) and weight change in relation to changes in ALM and hip BMD were correlated (0.40 < *r* < 0.74, *p* < 0.001).

**Table 2:** Pearson correlations between conditional change measures among men and women

Men	Grip strength	Gait speed	ALM	Fat mass	Hip BMD
Gait speed	**0.14**				
*p*-value	***< 0.001***				
ALM	**0.29**	0.07			
* p*-value	***< 0.001***	*0.072*			
Fat mass	**0.09**	0.05	**0.45**		
* p*-value	***0.020***	*0.234*	***< 0.001***		
Hip BMD	**0.27**	**0.24**	**0.36**	**0.29**	
* p*-value	***< 0.001***	***< 0.001***	***< 0.001***	***< 0.001***	
Weight	**0.19**	**0.09**	**0.73**	**0.90**	**0.41**
* p*-value	***< 0.001***	***0.024***	***< 0.001***	***< 0.001***	***< 0.001***
Women					
Gait speed	**0.17**				
* p*-value	***< 0.001***				
ALM	**0.20**	**0.08**			
* p*-value	***< 0.001***	***0.024***			
Fat mass	**0.12**	0.06	**0.46**		
* p*-value	***< 0.001***	*0.086*	***< 0.001***		
Hip BMD	**0.17**	**0.19**	**0.33**	**0.47**	
* p*-value	***< 0.001***	***< 0.001***	***< 0.001***	***< 0.001***	
Weight	**0.17**	0.07	**0.66**	**0.94**	**0.50**
* p*-value	***< 0.001***	*0.057*	***< 0.001***	***< 0.001***	***< 0.001***

*BMD* bone mineral density, *ALM* appendicular lean mass

Participants with at least two conditional change measures (735 men and 863 women) were included; pairwise correlations are displayed

Change measures were derived using a residual change method and are independent of baseline level

Significant correlations (*p* < 0.05) are in bold; correlations where *r* > 0.3 are in bold and underlined

### Sensitivity Analyses

Results were broadly similar between races as shown in the Supplementary Material. Racial differences in mean trajectories were larger regarding levels of the characteristics rather than in rates of change (eFigure 2 in Online Resource).

Participants with complete data had mean trajectories with slightly higher initial levels and/or somewhat lower rates of decline regarding grip strength, gait speed and hip BMD compared to participants with data at two or more time-points (eFigure 4 in Online Resource). However, the broad changes in the characteristics were similar among both groups of participants.

## Discussion

Among participants in the Health ABC Study, we have described, and examined associations between, changes in muscle strength, physical function and body composition parameters during a 9-year follow-up in later life. Declines in grip strength, gait speed and hip BMD accelerated somewhat with advancing age, whereas declines in ALM were linear. Declines were greater, and the proportion of variance at follow-up explained by baseline level was lower, for grip strength and gait speed in comparison with ALM, fat mass and hip BMD.

Our findings are consistent with those from previous analyses of the Health ABC Study. For example: over a 3-year follow-up, declines in knee extensor strength and both total and leg lean mass were correlated, although the absolute average magnitude of decline was greater for strength than mass [31]; 5-year declines in leg muscle torque were greater than those for muscle cross-sectional area [32]; and fat mass increased, plateaued and then decreased in a previous study of 5-year changes in body composition [33]. The latter suggests an initial trade-off between losing lean mass and gaining fat mass among weight-stable participants, followed by losses in weight, lean mass and fat mass with positive correlations between declines in weight and lean mass [34, 35]. Therefore, our analyses provide a longer term validation of these earlier findings.

Other cohorts have also described and compared longitudinal changes in muscle strength, physical function and body composition among older people. Our findings are in agreement with those from the Hertfordshire Cohort Study [20] and a cohort comprising 3018 Chinese participants, aged 65 and older [21], in which percentage declines in grip strength and gait speed were greater than declines in muscle mass. Although decline in grip strength curvilinearly accelerated with advancing age in the Health ABC Study, in a cohort comprising 8342 Danes (aged 50–85 years), linear declines in grip strength were observed [36] and in the Newcastle 85+ Study, grip strength decline was linear among men but quadratic among women [37]. A possible explanation for these conflicting results is the different age ranges and follow-up times of participants in these studies. Similar to our analyses, declines in grip strength and gait speed accelerated with advancing age among participants in the Cardiovascular Health Study [38] and this was also the case for total femoral neck BMD in the Osteoporotic Fractures in Men (MrOS) Study [39].

Previous studies have also examined interrelationships between changes in muscle strength, function and body composition. Changes in muscle strength and function in relation to changes in BMD among adults and children have been reported in a recent systematic review and meta-analysis [22]. Among adults, declines in BMD and the following characteristics were significantly correlated (*p* < 0.05): lean mass (*r* = 0.34 [95% CI 0.19–0.48]) [40–44]; grip strength (significant associations reported in all studies [45–47]); and gait speed [48]. Greater loss of arm lean mass was associated with accelerated loss of grip strength in a cohort of 1710 Afro-Caribbean men [49]. These findings are in agreement with our results from the Health ABC Study.

There are several potential mechanisms that may explain why longitudinal decreases in muscle mass, strength and BMD are correlated with one another in later life. First, the relationship between loss of muscle mass and strength may be bidirectional. Reductions in strength may result in declines in physical function and activity, leading to disuse-induced muscle wasting; simultaneously, declines in muscle mass and quality due to losses in fast-twitch muscle fibres, fat infiltration of skeletal muscle and increased inflammation may result in declines in strength and physical function [50]. Second, correlations between muscle mass and strength and BMD in older age are expected from both cellular and physiological perspectives. Cellular similarities include a shared mesenchymal stem cell origin between myoblasts and osteocytes [51]. Physiologically, reductions in strength lead to weaker forces on bone, resulting in greater bone resorption than formation according to the mechanostat theory [20, 52]. Finally, developmental, genetic, endocrine and lifestyle factors, such as smoking, physical activity and diet quality are established determinants of both muscle and bone aging [19], and may therefore contribute to correlations between declines in muscle and bone parameters.

A key strength of this study is the measurement of a wide range of musculoskeletal and body composition parameters in a single, well-characterised cohort. In contrast, studies which compare changes in musculoskeletal parameters across cohorts are likely to be limited because heterogeneous age ranges and nationalities of participants are likely to affect comparability of results. Another strength of the Health ABC Study is that parameters have been measured repeatedly over many follow-ups, enabling a comprehensive assessment of change.

This study has some limitations. Participants were free of mobility disability at baseline. This limits the generalisability of the findings to the wider population of community-dwelling older people in this age range and may have led to an underestimation of the magnitude of decline in these trajectories. Death and drop-outs during follow-up result in healthier participants remaining in the study who may be more likely to have slower rates of decline in musculoskeletal parameters. However, the inclusion of participants with two or more measures for examination of trajectories and calculation of percentage changes means that participants with short follow-ups are included in these analyses. Furthermore, the similarity of the trajectories for participants with observations at all time-points and those for participants with two or more repeated measures (eFigure 4 in Online Resource) suggests that drop-outs have only a small effect on the mean trajectories observed.

Our findings have important implications. Declines in later life were greater, and the proportion of variance at follow-up explained by baseline level was lower, for grip strength and gait speed compared with ALM, fat mass and hip BMD. This suggests that interventions that target body composition alone may be insufficient to also prevent the loss of muscle strength and function in this age group; these may require a broader range of intervention strategies, both to maximise peak levels in earlier life and to reduce age-related declines in later life. These findings may inform the development of lifecourse intervention strategies to prevent or delay adverse changes in musculoskeletal aging.

## Electronic supplementary material

Below is the link to the electronic supplementary material.

Supplementary file1 (DOCX 506 kb)
