# Supplementary material for: Long-term rates of change in musculoskeletal aging and body composition: findings from the Health, Aging and Body Composition Study
Source: Calcif Tissue Int. 2020 Mar 3;106(6):616–24. doi: 10.1007/s00223-020-00679-2 (PMC7188697; doi:10.1007/s00223-020-00679-2)
Supplement: Supplementary file 1 — Supplementary file1 (DOCX 506 kb) [file 223_2020_679_MOESM1_ESM.docx]

**Article title:** Long term rates of change in musculoskeletal aging and body composition: findings from the Health, Aging and Body Composition Study

**Journal:** Calcified Tissue International and Musculoskeletal Research

**Authors**: LD Westbury, HE Syddall, NR Fuggle, EM Dennison, JA Cauley, EJ Shiroma, RA Fielding, AB Newman, C Cooper

**Affiliations and e-mail address for corresponding author**:

MRC Lifecourse Epidemiology Unit, University of Southampton, Southampton, UK

NIHR Southampton Biomedical Research Centre, University of Southampton and University Hospital Southampton NHS Foundation Trust, Southampton, UK

NIHR Musculoskeletal Biomedical Research Centre, University of Oxford, Oxford, UK

cc@mrc.soton.ac.uk

**eFigure 1: Estimated annual percentage change in characteristics according to ethnicity and sex**

ALM: Appendicular lean mass; BMD: Bone mineral density

The three vertical lines in the box represent the lower quartile (Q1), median and upper quartile (Q3). The lower whisker is the smallest value that is greater than Q1 − 1.5 × IQR and the upper quartile is the largest value which is less than Q3 + 1.5 × IQR, where IQR = Q3-Q1.

Estimates of percentage change for each participant were derived using person-specific linear regression models for percentage change since baseline calculated at each time-point as the outcome with age at each time-point as the only predictor. Annual percentage change is given by the regression coefficient for age.

Analyses restricted to 1418 men (907 white and 511 black) and 1499 women (823 white and 676 black) with data on at least one change measure.

**eFigure 2: Mean (95% CI) trajectories of characteristics according to sex and ethnicity**

ALM: appendicular lean mass; BMD: bone mineral density

Mean trajectories were derived using linear mixed effects models with random intercepts and slopes. Quadratic and cubic age terms were included as fixed effects if significant (p<0.05)

For each characteristic, trajectories from participants with at least two observations were included

| **eTable 1: Pearson correlations between conditional change measures among men according to ethnicity** | | | | | |
| --- | --- | --- | --- | --- | --- |
| ***White men*** | **Grip strength** | **Gait speed** | **ALM** | **Fat mass** | **Hip BMD** |
| **Gait speed** | **0.19** |  |  |  |  |
| *P-value* | ***<0.001*** |  |  |  |  |
|  |  |  |  |  |  |
| **ALM** | **0.30** | 0.09 |  |  |  |
| *P-value* | ***<0.001*** | *0.050* |  |  |  |
|  |  |  |  |  |  |
| **Fat mass** | **0.11** | 0.01 | **0.43** |  |  |
| *P-value* | ***0.021*** | *0.754* | ***<0.001*** |  |  |
|  |  |  |  |  |  |
| **Hip BMD** | **0.31** | **0.26** | **0.38** | **0.29** |  |
| *P-value* | ***<0.001*** | ***<0.001*** | ***<0.001*** | ***<0.001*** |  |
|  |  |  |  |  |  |
| **Weight** | **0.20** | **0.06** | **0.74** | **0.89** | **0.42** |
| *P-value* | ***<0.001*** | ***0.20*** | ***<0.001*** | ***<0.001*** | ***<0.001*** |
|  |  |  |  |  |  |
| ***Black men*** | **Grip strength** | **Gait speed** | **ALM** | **Fat mass** | **Hip BMD** |
| **Gait speed** | 0.05 |  |  |  |  |
| *P-value* | *0.529* |  |  |  |  |
|  |  |  |  |  |  |
| **ALM** | **0.27** | 0.03 |  |  |  |
| *P-value* | ***<0.001*** | *0.690* |  |  |  |
|  |  |  |  |  |  |
| **Fat mass** | 0.06 | 0.11 | **0.49** |  |  |
| *P-value* | *0.390* | *0.158* | ***<0.001*** |  |  |
|  |  |  |  |  |  |
| **Hip BMD** | **0.19** | **0.19** | **0.31** | **0.31** |  |
| *P-value* | ***0.010*** | ***0.012*** | ***<0.001*** | ***<0.001*** |  |
|  |  |  |  |  |  |
| **Weight** | **0.18** | 0.14 | **0.73** | **0.91** | **0.40** |
| ***P-value*** | ***0.009*** | *0.055* | ***<0.001*** | ***<0.001*** | ***<0.001*** |
| *Note.* BMD: Bone mineral density; ALM: Appendicular lean mass  Men with at least two conditional change measures (518 white men and 217 black men) were included; pairwise correlations are displayed.  Change measures were derived using a residual change method and are independent of baseline level. | | | | | |

| **eTable 2: Pearson correlations between conditional change measures among women according to ethnicity** | | | | |  |
| --- | --- | --- | --- | --- | --- |
| ***White women*** | **Grip strength** | **Gait speed** | **ALM** | **Fat mass** | **Hip BMD** |
| **Gait speed** | **0.18** |  |  |  |  |
| *P-value* | ***<0.001*** |  |  |  |  |
|  |  |  |  |  |  |
| **ALM** | **0.21** | **0.09** |  |  |  |
| *P-value* | ***<0.001*** | ***0.047*** |  |  |  |
|  |  |  |  |  |  |
| **Fat mass** | **0.12** | 0.07 | **0.42** |  |  |
| *P-value* | ***0.009*** | *0.118* | ***<0.001*** |  |  |
|  |  |  |  |  |  |
| **Hip BMD** | **0.17** | **0.20** | **0.33** | **0.47** |  |
| *P-value* | ***<0.001*** | ***<0.001*** | ***<0.001*** | ***<0.001*** |  |
|  |  |  |  |  |  |
| **Weight** | **0.16** | 0.09 | **0.62** | **0.93** | **0.51** |
| *P-value* | ***<0.001*** | *0.068* | ***<0.001*** | ***<0.001*** | ***<0.001*** |
|  |  |  |  |  |  |
| ***Black women*** | **Grip strength** | **Gait speed** | **ALM** | **Fat mass** | **Hip BMD** |
| **Gait speed** | **0.20** |  |  |  |  |
| *P-value* | ***0.002*** |  |  |  |  |
|  |  |  |  |  |  |
| **ALM** | **0.19** | 0.07 |  |  |  |
| *P-value* | ***0.001*** | *0.240* |  |  |  |
|  |  |  |  |  |  |
| **Fat mass** | 0.13 | 0.05 | **0.51** |  |  |
| *P-value* | *0.028* | *0.439* | ***<0.001*** |  |  |
|  |  |  |  |  |  |
| **Hip BMD** | **0.22** | **0.16** | **0.33** | **0.47** |  |
| *P-value* | ***<0.001*** | ***0.011*** | ***<0.001*** | ***<0.001*** |  |
|  |  |  |  |  |  |
| **Weight** | **0.17** | 0.05 | **0.71** | **0.94** | **0.50** |
| *P-value* | ***0.002*** | *0.410* | ***<0.001*** | ***<0.001*** | ***<0.001*** |
| *Note.* BMD: Bone mineral density; ALM: Appendicular lean mass  Women with at least two conditional change measures (529 white women and 334 black women) were included; pairwise correlations are displayed.  Change measures were derived using a residual change method and are independent of baseline level.  Significant correlations (p<0.05) are in bold; correlations where r>0.3 are in bold and underlined. | | | | | |

**eFigure 3: Proportion of variance at follow-up (Year 10) explained by baseline level and conditional change since baseline according to sex and ethnicity**


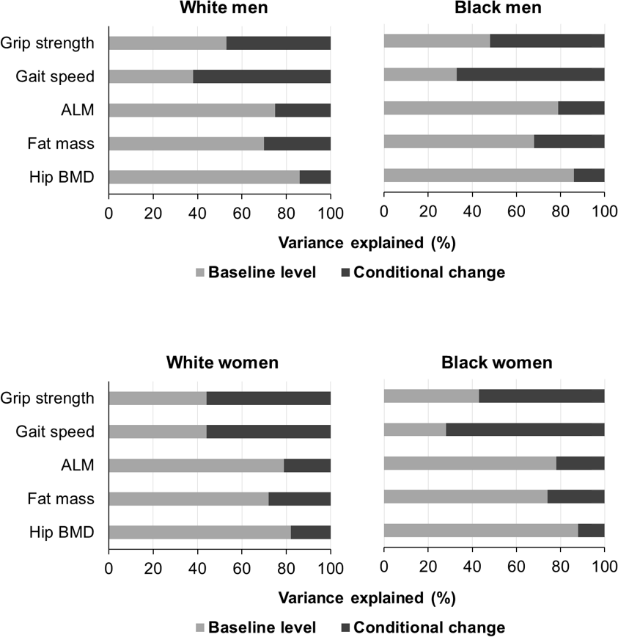


Measures of conditional change were derived using a residual change method and were independent of baseline level

Analyses restricted to 735 men (518 white and 217 black) and 864 women (529 white and 335 black) with data on at least one change measure

**eFigure 4: Mean (95% CI) trajectories among participants with data from at least two time-points compared to trajectories among participants with data at all time-points**

Mean trajectories were derived using linear mixed effects models with random intercepts and slopes.

Quadratic and cubic age terms were included as fixed effects if significant (p<0.05)
